# Implementing Automated Text Messaging for Patient Self-management in the Veterans Health Administration: Qualitative Study Applying the Nonadoption, Abandonment, Scale-up, Spread, and Sustainability Framework

**DOI:** 10.2196/31037

**Published:** 2021-11-15

**Authors:** Vera Yakovchenko, D Keith McInnes, Beth Ann Petrakis, Chris Gillespie, Jessica M Lipschitz, Megan B McCullough, Lorilei Richardson, Brian Vetter, Timothy P Hogan

**Affiliations:** 1 Center for Healthcare Organization and Implementation Research VA Bedford Healthcare System Bedford, MA United States; 2 Department of Health Law Policy and Management Boston University School of Public Health Boston, MA United States; 3 Department of Psychiatry Brigham and Women’s Hospital Boston, MA United States; 4 Harvard Medical School Boston, MA United States; 5 Department of Public Health University of Massachusetts Lowell, MA United States; 6 Office of Connected Care Veterans Health Administration Washington, DC United States; 7 Department of Population and Data Sciences University of Texas Southwestern Medical Center Dallas, TX United States

**Keywords:** implementation science, implementation facilitation, texting, veterans, eHealth, self-management, digital health, digital medicine

## Abstract

**Background:**

The Veterans Health Administration (VHA) is deploying an automated texting system (aTS) to support patient self-management.

**Objective:**

We conducted a qualitative evaluation to examine factors influencing national rollout of the aTS, guided by the Nonadoption, Abandonment, Scale-up, Spread, and Sustainability (NASSS) framework, which is intended to support the evaluation of novel technologies.

**Methods:**

Semistructured interviews were conducted with 33 staff and 38 patients who were early adopters of the aTS. Data were analyzed following deductive and inductive approaches using a priori codes and emergent coding based on the NASSS.

**Results:**

We identified themes across NASSS domains: (1) Condition: The aTS was considered relevant for a range of patient needs; however, perceptions of patient suitability were guided by texting experience and clinical complexity rather than potential benefits. (2) Technology: Onboarding of the aTS presented difficulty and the staff had different opinions on incorporating patient-generated data into care planning. (3) Value: Supply-side value relied on the flexibility of the aTS and its impact on staff workload whereas demand-side value was driven by patient perceptions of the psychological and behavioral impacts of the aTS. (4) Adopters: Limited clarity on staff roles and responsibilities presented challenges in incorporating the aTS into clinical processes. (5) Organization: Staff were willing to try the aTS; however, perceptions of leadership support and clinic readiness hindered usage. (6) Wider system: Staff focused on enhancing aTS interoperability with the electronic medical record. (7) Embedding and adaptation over time: The interplay of aTS versatility, patient and staff demands, and broader societal changes in preferences for communicating health information facilitated aTS implementation.

**Conclusions:**

VHA’s new aTS has the potential to further engage patients and expand the reach of VHA care; however, patients and staff require additional support to adopt, implement, and sustain the aTS. The NASSS highlighted how the aTS can be better embedded into current practices, which patients might benefit most from its functionality, and which aspects of aTS messages are most relevant to self-management.

**Trial Registration:**

ClinicalTrials.gov NCT03898349; https://clinicaltrials.gov/ct2/show/NCT03898349

## Introduction

Although use of virtual care technologies is expanding, automated text-messaging systems supporting patient self-management remain underused [1]. With the ubiquity of mobile phones, automated texting systems have the potential for wide reach and sustained use, representing a promising alternative to telephone calls, traditional mail, and emails [2-5]. Because of its simplicity, flexibility, and low cost, texting is increasingly recognized as a tool to reach those who may be less engaged in health care services and may thus narrow health disparities [6]. Studies show positive results for texting interventions, with demonstrated effects on chronic illness self-management, medication adherence, missed appointments, and behavior change, including weight loss and smoking cessation [2,7-10]. Despite promising evidence, the determinants of adoption, implementation, and sustainment of texting interventions are not well understood [11-15].

In 2016, the Veterans Health Administration (VHA) launched an automated texting system (aTS) called “Annie,” modeled after the Florence aTS developed by the United Kingdom’s National Health Service [16,17]. The aTS texting protocols are intended to promote, motivate, and enhance self-management by helping patients understand, track, and monitor their own health through 1- and 2-way messages. The aTS is condition-agnostic and able to support preprogrammed texting protocols for a variety of health conditions and behaviors. At present, there are over 100 aTS texting protocols available for use with VHA patients. These protocols fall into three categories: (1) nonpatient-specific protocols pertaining to conditions that do not require clinical diagnosis and treatment and those that patients registered with the aTS can self-subscribe (eg, tobacco cessation, coronavirus precautions); (2) nontreatment protocols intended to deliver educational and motivational messages, and generic reminders; and (3) treatment protocols that pertain to conditions requiring diagnosis and treatment by a clinician, and are intended to assist patients with self-management. Any VHA staff member regardless of licensure can register and confirm patient participation in the aTS as well as consent a patient to participate in a nontreatment aTS protocol; however, only licensed VHA clinicians can consent a patient for aTS treatment protocols. When initiating use, patients are asked to acknowledge that they understand the privacy implications of texting, which is not a secure or encrypted form of communication; they must understand that are texting with a computer system, and VHA staff may not regularly read or review the messages, making the system inappropriate for urgent issues or emergencies. Although VHA’s aTS was designed as a patient-facing self-management tool, clinical team members can choose to view patient message exchanges and track responses over time through a staff-facing aTS portal.

Some of the earliest aTS protocols addressed issues ranging from physical activity engagement, medication adherence, and blood glucose monitoring to colonoscopy preparation, diabetes management and foot care, hypertension management, smoking cessation, and weight management. More recent protocols have focused on COVID-19 precautions and vaccination support, HIV treatment, chronic pain, insomnia, and maternity care. Staff can tailor protocols (eg, content, periodicity, timing) based on patient needs and preferences. A multidisciplinary group of VHA content and technical subject matter experts maintains the aTS protocol library and guides staff from across VHA facilities on how to adapt existing protocols, and to create, test, and implement new protocols. In an implementation-effectiveness cluster randomized trial of the aTS, Yakovchenko et al found that test sites with enhanced aTS implementation support not only had more patients using the aTS, but these patients also reported better adherence to treatment and lower distress about failing treatment compared to usual aTS implementation sites [1].

This qualitative evaluation examines the implementation experiences of early aTS adopters in VHA to inform national rollout of the system and improve its design and functionality. Findings from this evaluation can provide other health care systems with an understanding of the implementation challenges they might face when introducing and expanding their own texting systems.

We used the Nonadoption, Abandonment, Scale-up, Spread, and Sustainability (NASSS) framework to organize and synthesize our findings [18,19]. The NASSS framework identifies 7 domains influencing the uptake and use of patient-facing technologies: condition, technology, value proposition, adopter system, organization, broader surrounding context, and interaction among these domains. An advantage of a complexity-informed framework like NASSS is its acknowledgment that complex adaptive systems develop and behave in unpredictable, dynamic, and nonlinear fashions.

## Methods

### Design

We used a qualitative interpretive phenomenological approach to explore staff and patient experiences with VHA’s aTS. Semistructured interviews were conducted between June 2016 and February 2018 as part of a larger evaluation of the implementation and effectiveness of the aTS. Verbal consent was obtained prior to interviews. The VHA Bedford Healthcare System Institutional Review Board reviewed the evaluation and determined it to be a program evaluation for quality improvement purposes, thereby exempting it from further Institutional Review Board oversight (VHA Program Guide 1200.21). The study was registered at ClinicalTrials.gov (NCT03898349).

### Setting and Participants

We conducted semistructured interviews with purposively sampled participants from 14 VHA medical centers. The sites represented diversity in geography, rurality and urbanicity, patient volume, sample size, and complexity. Staff participants were VHA clinical team members (hereafter referred to as staff), including physicians, nurses, dieticians, pharmacists, and social workers. Patient participants were individuals who received health care services at these sites.

### Data Collection

Staff interview guides focused on the staff perceptions regarding the aTS, implementation barriers and facilitators, setup and enrollment procedures, implications for care delivery, clinical workload, patient-provider relationship, and experiences using the system. Patient interview guides explored the patient perceptions of the aTS, including its influence on relationships with staff and engagement in care, usefulness of the aTS for supporting self-management, and factors that might influence use and perceived usefulness. Interview guides were reviewed by VHA’s aTS designers for clarity, pilot-tested among the evaluation team members, and iteratively revised.

Interviews were conducted in person at VHA medical centers and over telephone, typically lasting up to 60 minutes. We gathered participant demographic data immediately prior to the start of the interviews using a brief questionnaire. Most interviews were one-on-one. In some instances where 2 staff members were interviewed simultaneously to accommodate availability, a primary interviewer led the discussion and a secondary interviewer assisted and took field notes. There were 3 male and 3 female interviewers (DKM, BAP, CG, JML, MBM, and TPH) who were masters- and doctoral-level public health, public administration, psychology, and anthropology professionals. Reflexivity was considered during the interviews and throughout the evaluation. All interviews were audio-recorded and transcribed verbatim. Patients received a gift card of a local store as compensation for their time. Staff were not eligible for compensation due to VHA regulations.

### Analysis

Descriptive statistics were used to characterize participant demographics. Interview data were analyzed using deductive and inductive approaches with the NVivo 12 Pro software (QSR International). Coding and analyses were performed by 6 trained masters- and doctoral-level researchers with extensive qualitative research experience (VY, DKM, BAP, CG, LR, TPH). We drew upon the NASSS domains to create a preliminary codebook, and additional codes were inductively added if they were not otherwise reflected in the framework. A subset of interviews was examined by all evaluation team members to formulate coding rules through a process of critical review and consensus building. Recurring meetings were held to compare coder interpretations and discuss coding discrepancies. As the final step, we used the NASSS complexity assessment tool (CAT) to inform categorization of each domain as simple (straightforward, predictable, few components), complicated (remains predictable but with multiple interacting components or issues), or complex (dynamic, unpredictable, not easily disaggregated into constituent components) [20].

## Results

### Participant Characteristics

We conducted a total of 71 interviews with 38 VHA patients and 33 VHA staff representing a range of experiences with the aTS, as shown in Table 1. Most patients were male (n=30, 80%) and White non-Hispanic (n=29, 76%), with a median age of 56 years. Nearly all had a smartphone (n=37, 97%) and texted daily (n=30, 79%). Most also used computers (n=33, 86%) and the internet (n=34, 90%) daily. However, patients had varying experiences with VHA’s aTS, with some having only registered with the system, others having received 1-way messages, and others having sent and received daily messages over multiple months. Among staff, 63% (n=21) were clinical (MD, NP, RN), 20% (n=7) were clinical pharmacists, and 22% (n=7) were other types of staff (eg, dietician, social worker). Most were women (n=25, 76%), with a median age of 36 years and 8 years of VHA work experience. Most staff (n=27, 81%) used mobile phones several times a day to send or receive messages. At the time of the interviews, staff reported varying experiences with the aTS, including some who had used the aTS from 1 day to 6 months and had enrolled 0 to over 20 patients.

**Figure 1:**
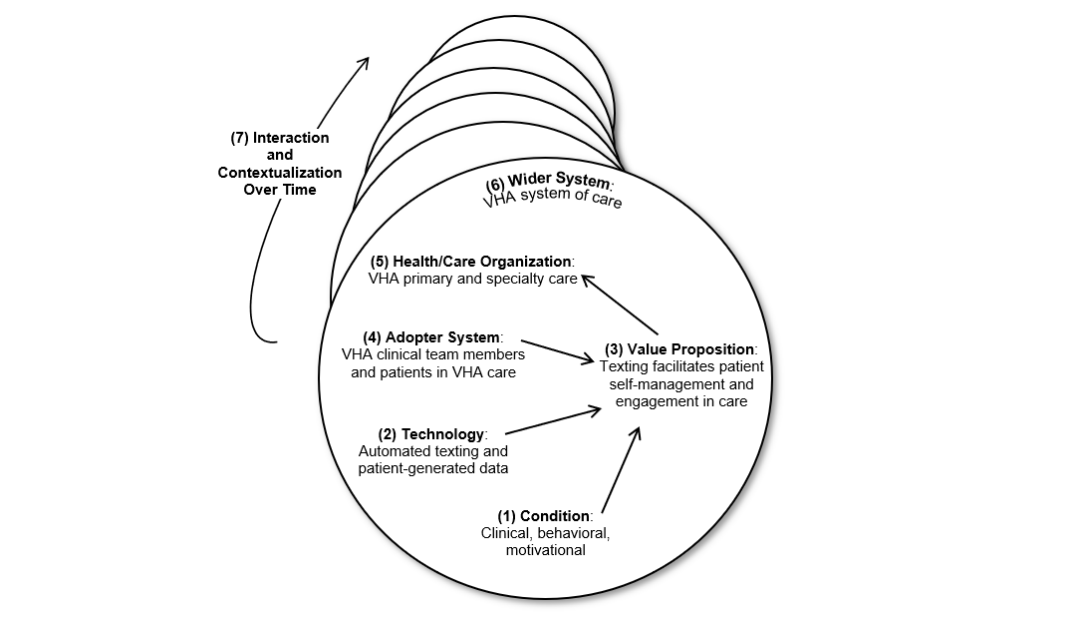
Nonadoption, Abandonment, Scale-up, Spread, and Sustainability framework in the Veterans Health Administration (adapted from Greenhalgh [19]). VHA: Veterans Health Administration.

We present our findings organized based on the NASSS framework domains, as displayed in Figure 1 and Table 2. Quotes are attributed to patients (pt) or staff (s).

**Table 1:** Participant characteristics.

Participants and characteristics			Value, n (%)
**Patients (N=38)**			
	Median age, years		59
	Male		32 (85)
	Race/ethnicity: White/non-Hispanic		26 (68)
	Daily texting		30 (79)
**Staff (N=33)**			
	**Staff type**		
		MD^a^, NP^b^, RN^c^	21 (64)
		PharmD^d^	5 (15)
		Other (dietician, social worker)	7 (21)
	Median age, years		40
	Median VHA^e^ tenure, years		8
	Male		8 (24)
	Daily texting		28 (84)

^a^MD: Doctor of Medicine.

^b^NP: nurse practitioner.

^c^RN: registered nurse.

^d^PharmD: Doctor of Pharmacy.

^e^VHA: Veterans Health Administration.

**Table 2:** Themes and quotes based on Nonadoption, Abandonment, Scale-up, Spread, and Sustainability domains.

NASSS^a^ domain	Theme	Provider quote	Patient quote
Domain 1: Condition or illness	Patient suitability	“The patient population here tends to really need a lot of hand holding and a lot of TLC^b^.”	“…made the treatment a heck of a lot easier, because I remembered to take the pills on time.”
Domain 2: Technology	Knowledge, support features, and functionality; patient-generated data	“It took me a little while to familiarize myself because the training versus actually doing it yourself, you know, there’s a learning curve…”	“It meets my needs and probably in the future, as my conditions change or my needs increase, it will be there.”
Domain 3: Value proposition	Supply-side and demand-side values	“We’re actually really excited to use it with our patients. This is something that we had talked about doing or developing something like this…”	“…would tend to get a little overwhelmed at times, but Annie helped alleviate that.”
Domain 4: Adopter system	Staffing, roles, and skills; complex decisions	“I thought a lot of people are going to be able to participate but I guess when we started offering…some patients don’t, I guess they’re not used to it, most the patients that I offer decline to participate.”	“I don’t even know who would be doing it. Would it be the doctor? Or the nurses, or? Whose responsibility would it be to actually implement that? So, I don’t know how practical that would actually be, but I think it would be better if they did gear it more toward that individual patient and whatever problems they’re having.”
Domain 5: Organization	Leadership and readiness to innovate; workflows and routines	“Whenever you’re using new technology and new approaches with the technology component it’s just good to have somebody that you can, who’s very responsive and can find out the answer for you in a timely fashion…”	“And my understanding was this was to alleviate a lot of paperwork. They just send me a text message and that way I can write it in my date book or keep it in my phone till the time for the appointment.”
Domains 6 and 7: Wider context and embedding; adaptation over time	Fiscal interoperability, digitalization, and marketing	“But in order for this to work this has to be easy for the provider and easy for the Veteran. Otherwise, it’s not gonna work, it’s not gonna help, we’re not going to be effective.”	“I use Annie for other medications as well…I just group them all in together.”

^a^NASSS: Nonadoption, Abandonment, Scale-up, Spread, and Sustainability.

^b^TLC: tender loving care.

### NASSS Domain 1: Condition

Staff described the aTS as a tool to support patients in activating and maintaining health-related behaviors across various conditions and care contexts. The staff and patients described the relevance of the aTS to a range of needs, including multidose vaccinations, HIV prevention and care, birth control, breast cancer screening, chemotherapy, dialysis, postoperative support, mindfulness, yoga, and anxiety management. For some, the options at times could feel overwhelming. The aTS protocols for disease processes that are “pretty well mapped out” were especially appealing to staff, as were the protocols for medication management, appointment reminders, and laboratory reminders before appointments. Patients believed the aTS could “start the habit” (pt140) to activate health behavior change and influence patterns of self-care.

#### Patient Suitability

Although staff were using the aTS under different conditions, they expressed similar perspectives on patient suitability to use the system. Although they generally understood that the aTS was “open to pretty much anybody,” considerations about who might be appropriate for the aTS converged around several preconceived, largely nonclinical criteria that informed their offering of the aTS to patients. These criteria included patients being younger in age, perceived to have greater texting savviness and technological literacy, and a higher baseline motivation to change. Staff preferred to engage patients whom they predicted would agree to use the aTS and to avoid having conversations about the aTS with others: “…there’s no sense in even bringing (the aTS) up because it just wouldn’t be for them” (s224).

Staff further emphasized that they thought the aTS could complement traditional care for patients who might be prone to neglecting their own health or are facing various health and social challenges like memory issues and other cognitive impairments, limited social support, homelessness, and low health and technological literacy, with whom they must “work creatively” to engage in care. For more complex patients, an option was to consider caregivers as potential recipients of aTS messages. Patients and staff emphasized the importance of patients being motivated and having baseline self-efficacy to benefit from the aTS: “If you want to do it, be serious about it,” (pt120, male 50 years old). Staff shared their observations that once patients started using the aTS, they tended to continue using it, and attrition was low. Patients expressed varying levels of interest in the system, from those who were actively interested (“I want it to be that thing overlooking my shoulder that gives me a little extra discipline,” [pt124]) to those who were simply not interested at all (“I have no desire to play with the telephone,” [pt119]). Several staff shared their concern that some patients would agree to use the aTS to appease them during an in-person appointment and then not follow through once the protocol started. Staff mentioned needing support in “finding the correct patients” (s224). One staff member suggested a targeted recruitment approach to address issues of patients who were struggling. They suggested, “look for outliers with high blood pressure, outliers with diabetes, so they could easily focus on people and they can sell it” (s205). However, such a clinically focused patient outreach for the aTS was less commonly described than the practice of targeting patients based on the nonclinical characteristics noted above.

### NASSS Domain 2: Technology

**Figure 2:**
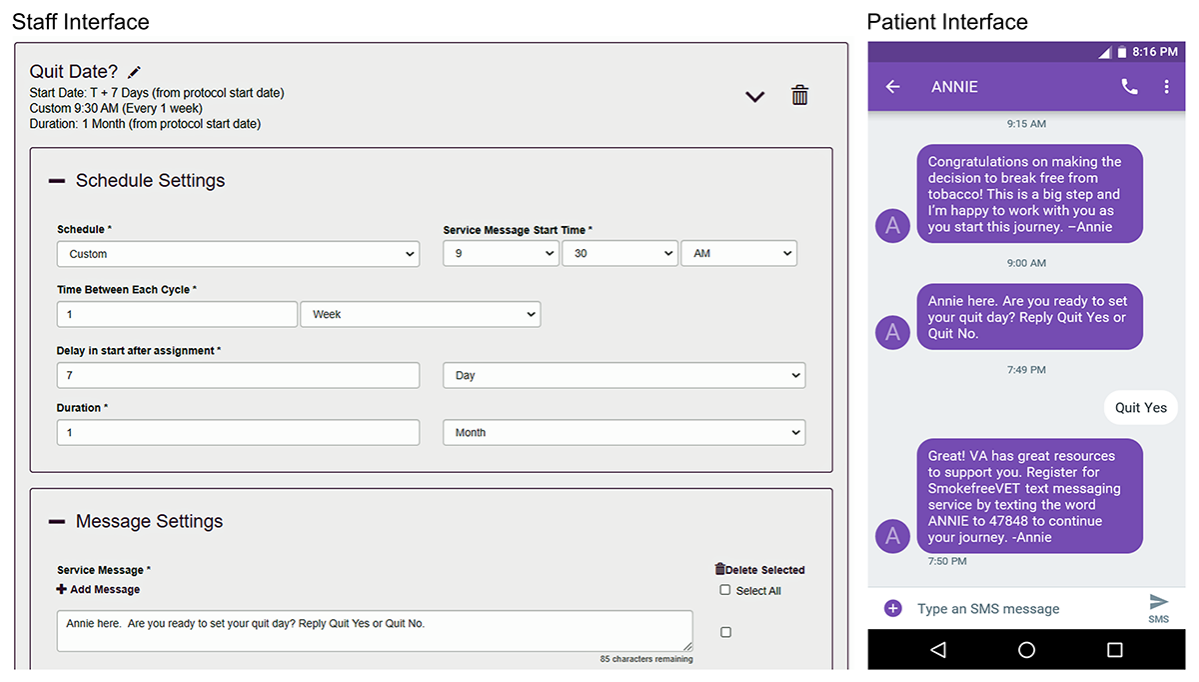
Screenshots of the staff and patient interfaces of the automated text messaging system for tobacco cessation protocol.

The aTS has patient- and staff-facing components that differ in terms of their interface, functionality, and complexity. The left panel in Figure 2 displays the aTS interface as seen by staff when assigning protocols, whereas the right panel displays a text exchange as seen by patients.

#### Knowledge and Support

Because staff invited patients with previous texting experience to use the aTS, there was no pressing need for staff-to-patient training. Conversely, staff acknowledged a steep learning curve that influenced their willingness to use and continue using the aTS, even with training, access to written materials, and live support from the aTS designers. Many commented about the time lag between training and the first enrollment of patients, which necessitated refresher training and technical support. However, once comfortable with the aTS, many deemed it “fairly straightforward and seamless” (s303), although some still found it “clunky” and “a little bit slow.”

Perceptions on the time needed to introduce, consent, and enroll patients in the aTS were strong deterrents of use for busy staff. The process of signing into the system and assigning a protocol to patients was considered complicated and a point at which patients may lose interest or get frustrated; however, once this front-end “logjam” of tasks was completed, demands on staff time were manageable. One nurse practitioner commented that “my efficiency of enrolling (patients) and following through makes a big difference in time…(once) you’re comfortable with (the aTS) you are more apt to continue to do it because you know it’s not going to take you a lot of time” (s312). Several staff suggested that the aTS designers must consider opportunities to automate enrollment, perhaps through other existing technologies like waiting room kiosks, to bypass the extensive front-end tasks.

#### Features and Functionality

Regarding functionality, many patients reported difficulties with the proper syntax required to send responses to the aTS (eg, incorrectly responding “Yes” instead of “Med Yes”). Although some learned to use the correct syntax as indicated in the aTS messages, a few abandoned the system out of frustration. However, most patients commented on the simplicity of the aTS: “Easy to use, it was no problem…quick and to the point” (pt318). Some patients struggled to understand that the aTS was not a direct line of communication with their clinical team (ie, unlike secure messaging through VHA’s online patient portal). Several patients indicated that they were interested in the ability to text staff directly, but the staff overwhelmingly did not want such a functionality.

The ability to tailor aspects of the aTS interaction was considered a positive system feature among patients and staff, although there were some who were unaware this was possible. The potential to create texting protocols and tailor messages for individual patients enhanced the staff’s sense of ownership and perceived value of the aTS. Some staff saw the process of tailoring the aTS as shared decision-making with their patients, and others appreciated the system’s support of patient autonomy, allowing patients to use the aTS when they want to engage. As one physician noted, patients might experience texting fatigue. “The patients can pause it if they need a break. And it might be that they pause and 2 weeks down the road they can reactivate it” (s214).

#### Patient-Generated Data

There were mixed opinions among staff on how best to use patients’ aTS responses. The patients and staff were interested in visualizing patients’ numerical responses to the aTS messages over time, such as blood pressure and weight values. Staff appreciated the ability to longitudinally record such data in between visits but lamented that real-time data were not recorded directly in the electronic medical record (EMR). Some staff were less concerned about transferring this patient-generated data to the EMR and embraced reviewing the aTS portal’s message dashboard, occasionally together with patients. Reviewing a patient’s message logs prior to an appointment was considered helpful to guide conversation, titrate medication, and revise health plans. However, others were opposed to reviewing patient messages, yet another source of information to manage, as they believed the aTS fell entirely in the realm of self-management and was not intended for staff monitoring. As for those with more favorable opinions of the patient-generated data, some staff were unclear how, if at all, to use it: “What am I going to do with that information? Am I going to schedule an appointment with the patient? Am I going to call the patient, which is now extra work?” (s224).

### NASSS Domain 3: Value Proposition

#### Supply-Side Value

Staff perceived the aTS as versatile, amenable to supporting simple (eg, 1-way education and motivation), complicated (eg, 2-way appointment and lab reminders), and complex (eg, procedure preparation) messaging. The aTS was viewed as a welcome complement to education provided during in-person visits and offered patients flexibility to interact with, reflect on, and apply new learning about their health at their preferred pace. Staff felt the system was aligned with VHA’s larger goals of empowering patients, promoting self-management, and staying connected with patients between visits. Nevertheless, for some, perceptions of the workload associated with the aTS impeded their interest in using it.

Staff indicated several benefits of using the aTS. First, some stated the aTS was suitable for younger patients, who often prefer technology-mediated communication. Second, they recognized that investing in the aTS might have administrative benefits such as reducing appointment no-shows and increasing patient preparation for appointments (eg, necessary lab tests not completed). Third, there may be workload improvements from reducing repetitive clinical tasks. Such changes could enable other clinical team members to work at the top of their licenses. As one physiologist noted, the aTS “would open up time for the nurses to really focus their energy on patients who need a lot of tender loving care instead of just doing reminder phone calls on a weekly basis to track down patients…” (s206).

#### Demand-Side Value

Patients endorsed value in several areas. First, patients felt the aTS supported new learning about their health and saw the aTS as a means of promoting closer connections to their clinical team. As one patient commented, “It’s kind of like the little guy whispering in your ear that this is important. Your doctor is concerned about what you’re doing” (pt144). To this end, patients reported aTS messages were more credible compared to those that might come from other non-VHA technologies. Second, patients reported that texting had a psychological and behavioral impact, making them feel accountable, safer, and more comfortable. One patient who was participating in a VHA weight management program commented, “It’s eye opening to me and forces me to be honest with myself about the things I’ve done and the things I’ve eaten, so that’s a huge benefit because without having to do that every day it’s very easy to just mindlessly go on from day to day without thinking about it specifically” (pt122). Third, patients felt texting was desirable because it offers more control when one wants to absorb information. As one patient described, “You can accept a text message when you’re ready on your terms. That kind of stuff is not quite as invasive as a phone call…I think it’s a great way to present nonthreatening education, reminders, guidance” (s214). Finally, patients reported leveraging the aTS messages to support their self-management efforts in unintended ways. For example, reminders from the aTS to take one’s morning medications for a specific condition were used in practice by some patients as a reminder to take all their morning medications.

### NASSS Domain 4: Adopters

The characteristics and experiences of the intended aTS adopters, the staff and patients, constituted the most complex domain in our analysis, largely owing to ill-defined staff roles and responsibilities during system implementation and persistent concerns about patients’ understanding of self-management.

#### Staffing, Roles, and Skills

Despite having learned about the aTS functionality, many staff reported feeling unprepared to implement it after 1 training session, citing insufficient guidance on how to practically incorporate the aTS in their clinic and in-patient care planning. There was consensus that staff roles and interdependencies would need to change to accommodate aTS uptake and use, and many noted that without dedicated personnel, the aTS might have limited success. Staff recommended that one or several individuals, rather than an entire team, assume aTS responsibilities to ensure greater uptake and minimize disruption. Others proposed distributing enrollment steps across staff to promote teamwork.

Readiness and willingness to implement the aTS differed across staff. Pharmacists and nurses conveyed a higher readiness than physicians, many of whom were resistant. Some staff cautioned that having physicians play a central role would be a “rate-limiting step” in the system’s spread. As one physician commented, “If providers are expected to have a lot of involvement with this, it's gonna flop” (s211). Specifically, physicians sought a degree of separation from aTS education and enrollment processes, suggesting other staff as more appropriate for these tasks. However, many nurses saw the aTS as an extension of their work, including one who commented that the aTS “harmonizes perfectly” with their clinical role (s215). Staff described how nonlicensed medical support assistants could contribute to aTS enrollment processes after verbal consent was obtained from a licensed clinician. Finally, staff suggested that champions, such as existing telehealth coordinators, could help “unburden the staff from having to be the tech expert” (s217).

#### Complex Decisions

Staff often expressed hesitation about using 2-way texting because of concerns about interpreting and acting on the content of the patients’ replies (ie, reporting high blood pressure). Although patients were made aware during consent that messages were unmonitored, staff remained concerned about liability.

Patients voiced a parallel concern about not knowing who was overseeing the messages they exchanged through the aTS and the extent to which those messages reflected an understanding of their unique health situation. As one patient remarked, “Don’t get me wrong, but I don’t want a clerk sending these kinds of messages where a health provider should at least be seeing it” (pt111).

### NASSS Domain 5: Organization

According to the participants, the organization domain included various factors impacting aTS uptake, including leadership, readiness to innovate, and logistics of workflows and routines.

#### Leadership and Readiness to Innovate

At the time of executing this project, publicity for the system was highly localized, with no coordinated plan to raise awareness across leadership levels, stakeholders, or other staff. Most staff who tried the aTS were keen to learn about it and did so without a wider team or organizational involvement, citing a “willing (ness) to try new things” and openness to “new technology to help us with the Veterans” (s302). Staff cautioned that the leadership was “very contemplative,” “skeptical,” and required strong evidence to buy into new technology like the aTS. Although leadership buy-in was initially described as a “stumbling block,” most staff felt that with sufficient evidence, the aTS would with time become “an easy sell” (s216). Notably, clinic willingness to use the aTS varied within facilities, and staff at smaller facilities tended to view the aTS as a potential time-saving tool given their limited workforce. Interfacility variation stemmed from leadership and readiness, whereas intrafacility (between clinics within a facility) variation was more adopter-oriented.

#### Workflows and Routines

Staff uniformly recommended that the most important consideration should be “how are we going to work this into our flow” (s217). Staff were conflicted, noting that although the aTS is “a good thing for the patient…I don’t see where in the workday we can be checking this” (s203). Others expected that the aTS could offset some more administrative activities: “It would open up time for the nurses to focus their energy on patients who need a lot of tender loving care…to help streamline their work, so they could use their clinical skills more effectively” (s206). Nevertheless, at the time of our evaluation, facility-wide shared visions of how best to use the aTS were lacking, as were ideas regarding how best to coordinate aTS use to support its broader spread.

### NASSS Domain 6: Wider System, Interaction Between Domains, and Adoption Over Time

Wider sociocultural forces including financial, political, legal, and regulatory factors posed hurdles to aTS uptake and sustained use. The system’s long-term adoption and use was related to its design and flexibility, as well as the ability of staff, clinics, and facilities to monitor and respond to the system.

#### Fiscal

Staff wanted to be recognized for the time they were devoting to the aTS as well as be held accountable for and have dedicated time to use it with patients. However, staff were neither able to track workload credit to account for time spent on the aTS nor externally incentivized to use it.

#### Interoperability

Staff perceptions on the scale-up, spread, and sustainability of the aTS were largely based on two key system characteristics: (1) interoperability with the EMR and (2) potential to be used in conjunction with other existing technologies. Because of limited interoperability with the EMR, if staff wanted to include information in an aTS protocol specific to a patient (eg, appointment or medication refill dates), they had to manually enter the information into the protocol. This was not only laborious, but it also raised concerns about accuracy problems, particularly in the case of longer and more complex aTS protocols. For these reasons, some staff viewed the aTS as unsustainable. Some described the aTS as a “step down” from more intensive, clinician-directed initiatives such as VHA’s MOVE! weight management program, and its home telehealth program with remote monitoring and case management services for chronic health conditions. One dietician commented that the aTS could help patients “to still be aware of their health goals but not so dependent on us as clinicians to really be involved in that care” (s210).

#### Digitalization and Marketing

Patients and staff alike viewed the aTS as part of a larger nationwide digitalization initiative. Staff suggested national-level marketing to improve the visibility of the aTS and reduce the educational burden at the clinic level. Staff likened the aTS to the rollout of VHA’s online patient portal nearly 20 years earlier, suggesting that there may be transferable lessons. There was recognition that younger patients may be more amenable to adopting the aTS than the patients most staff were currently seeing for care, and although many younger patients may not yet be facing health problems, a focus on health promotion and disease prevention could help spread aTS use.

## Discussion

### Principal Findings

Guided by the NASSS, we evaluated the implementation of an aTS in the nation’s largest integrated health care system. The perceived value of the aTS derives from its versatility, patient and staff demand, and growing societal comfort and familiarity with technology that aids health-related communication. The aTS has considerable potential to complement traditional, in-person care as well as usage of other patient-facing technologies. Nevertheless, implementation posed challenges related to the system’s limited functionality, mixed user experiences, inadequately defined workflows, and limited interoperability with other systems like the EMR.

Although staff became competent in using the aTS, many were unable to integrate the system into their workflow given the limited duration of patient visits, technical challenges, and distribution of other clinical tasks. Staff appeared to assume that once a critical number of patients started using the aTS and the burden of educating, consenting, and enrolling large numbers of patients had passed, they would thereafter have the relatively easy task of assigning protocols. Despite being a plausible scenario, this can place a heavy burden on early staff adopters who may be expected to perform extra short-term work in the belief they will receive long-term benefits.

VHA and other health care systems are investing in technologies for remote delivery of health care services, and texting systems are arguably one of the most efficient forms of communication. However, we found that staff assumptions about many of their patients precluded universal offering of the aTS. Moving forward, safeguards must be established to protect against such bias. More targeted patient outreach was recommended by some participants and is supported by literature [21]. Ways to increase aTS use despite staff concerns could include streamlining the aTS enrollment process by offering the system to all new patients at the time of VHA health care enrollment and providing specially trained staff for this, thereby removing clinical staff from time-consuming front-end processes.

We found that some patients were skeptical about texting, whereas others felt little need to improve their self-management and therefore declined the aTS. Our data highlight how the process of tailoring aTS message content and timing not only encouraged patient use but also enhanced patient autonomy, which aligns with patient-centered care principles [22,23]. Although many staff reported that the responses patients sent to the system would rarely change the care they provided, most of the staff wanted access to the patient messages within the aTS and related system reports [24]. As the potential value of patient-generated data grows, future efforts may involve using the aTS to gather patient-reported outcomes, including satisfaction, comprehension of instructions, and postdischarge follow-up assessments [25].

In cases of modest patient interest in the aTS system as well as patient memory and cognitive issues, patients and staff suggested that informal caregivers might be greater beneficiaries of the aTS than patients. Indeed, Wagner et al found text messaging caregivers directly was significantly associated with changes in diabetes outcomes for patients [26]. Extending the reach of the aTS to caregivers requires further study as an implementation strategy and an approach to improving patient outcomes. Moreover, given the heterogeneity of past texting studies, more research is needed to determine associations between texting intervention characteristics (eg, frequency, timing, duration, interactivity) and outcomes [2].

The NASSS is quickly becoming a prominent meta-framework for identifying complexities and their interactions in studies of technology implementation [27,28]. The framework posits 3 levels of complexity—simple, complicated, and complex—which may predict technology adoption and nonadoption. New tools, including the NASSS-CAT, are particularly useful in explaining our data and could help guide technology implementation initiatives [20]. We used the NASSS-CAT retrospectively; future implementation work may consider using such tools prospectively over the duration of a project.

From our application of the NASSS framework, we determined that overall, the aTS was perceived as easy to use by patients (simple in NASSS terminology) and difficult by staff (complicated in NASSS terminology), a situation that could threaten scalability. In our analysis, the aspect most likely to hamper the uptake and spread of aTS was related to adopters (characteristics and experiences of intended users), principally because there was insufficient staff training, and staff roles and responsibilities for implementation tasks were poorly defined. We determined it as the most unpredictable and abstruse domain (complex in NASSS terminology). The only other complex domain was value (users’ perceived benefit of the technology), rated as such because staff could detect the potential of the aTS but could not always detect a relative advantage given workload demands. Such staff views likely fueled their doubts that aTS would be sustainable. Several NASSS domains presented moderate difficulty to staff and patients (ie, they were rated as complicated). These were condition (health issue being addressed), technology (innovation characteristics such as system usability and data generated), organization (characteristics of the health care system), and wider system (societal elements such as political and regulatory concerns). A frequently cited challenge was the lack of the interoperability of the aTS with the EMR and existing patient-facing technologies. Interoperability helps drive spread and sustainability by facilitating documentation of aTS enrollment, easily transferring patient responses to clinical notes, and autopopulating text messages unique to an individual patient’s situation. Integrating the aTS into the EMR has implications for workflows, potentially reducing staff burden. There may also be opportunities for integration of the aTS into population health management tools that are commonly used in VHA [29-32]. It is not surprising that no domains were rated as simple given the aTS is a dynamic platform that can address the needs of a range of health conditions, behaviors, and patients.

The challenges noted above need not hamper the uptake and spread of the aTS, and since the completion of this evaluation, VHA has made iterative refinements in the aTS, some of which address these challenges. A variety of practices can help with implementation of new technologies in large complex health care systems: (1) At the facility level, local champions can be identified or assigned, who can assist with multiple aspects of text-messaging promotion, such as marketing and education, technical assistance, and work groups and communities of practice [33]. (2) At central levels, an expert panel can be created to oversee text-messaging protocol governance, and, depending on its expertise and capacity, such a panel could also work with individual facilities to develop and test new protocols. (3) Marketing and educational efforts can be effective in producing materials, supporting websites and helplines, creating training platforms, and convening a community of practice. (4) Finally, dashboards can be created to track new enrollment in text-messaging systems in real time at the national, regional, and facility levels.

### Strengths and Limitations

One strength of this evaluation is the identification of diverse perspectives of VHA patients and staff from a range of disciplines and VHA settings. VHA is unlike many other health care systems, and veterans differ from nonveteran patients; therefore, our findings may not be entirely generalizable to other health care systems and patient populations. It is important to note that at the time of our work, the aTS was in a beta testing stage, and the system was actively being updated. In addition to impacting the user experience, staff who engaged with the system at this time could generally be considered early adopters and may differ from users who encounter the aTS at later stages of its implementation. Data were collected in 2 waves over a period of 1.5 years. We used the NASSS-CAT retrospectively, thus likely not taking full advantage of its potential. Future implementation work may do well to consider using such tools over the duration of a project prospectively and retrospectively.

### Conclusions

This is the first paper to report qualitative findings from the perspectives of patients and staff on factors affecting the adoption, implementation, and spread of VHA’s new aTS. NASSS, a meta-framework for identifying complex elements and their interactions, was an important tool to help classify these factors and recognize their interplay [27,28]. As health care systems implement new technologies to deliver high-quality, effective, patient-centered care, the multilevel complexities of adoption (or nonadoption), implementation, and sustainment must be studied. Insights gained from such evaluations continue to inform improvements in VHA’s aTS system and its national rollout and use, and they can aid in the scaling of texting interventions in other health care systems.

## Abbreviations

aTS: automated texting system
CAT: complexity assessment tool
EMR: electronic medical record
NASSS: Nonadoption, Abandonment, Scale-up, Spread, and Sustainability Framework
VHA: Veterans Health Administration
